# Treatment of a Giant Fusiform Basilar Aneurysm with Partial Intra-Aneurysmal Embolization Combined with Mid-Basilar Artery Occlusion in a Child

**DOI:** 10.1007/s00062-015-0451-6

**Published:** 2016-04-26

**Authors:** J. You, Z. Ma, F. Zhang, G. Li

**Affiliations:** Department of Neurosurgery, Cerebrovascular Center, the Second Affiliated Hospital, Guangzhou University of Chinese Medicine, 111 Dade Road, 510120 Guangzhou, Guangdong P.R. China

## Introduction

Intracranial aneurysms are rare in the pediatric population, defined as patients 18-year-old or younger, with a reported prevalence ranging from 0.5 to 4.6 % [1–5]. Recent studies demonstrate that 17–27 % of these aneurysms are on the posterior circulation and 20–45 % are giant [6–8]. Aneurysms of the basilar artery (BA) trunk pose difficult therapeutic challenges. In the neurosurgical literature, these aneurysms are poor regarding their natural history and outcome, as they are associated with 80 % mortality [9]. Different complex skull base approaches have been attempted to access the basilar trunk area using several techniques like temporary balloon occlusion [10] or even with the aid of hypothermic cardiac arrest [11]. This has prompted the neurointerventional endovascular approaches to treat posterior circulation aneurysms. Recent reports have documented successful results using electrolytically detachable coils with or without assisted balloon or stent techniques in the treatment of many posterior circulation aneurysms [12–15]. Selective occlusion of the aneurysmal sac is the choice. However, there are some cases where selective aneurysm obliteration by either surgical clipping or endovascular approach is impossible or associated with an unacceptable risk of morbidity. This is particularly true when the aneurysm is giant or has a large neck or involves an eloquent perforator area like BA trunk area [16–18]. In such cases, additional parent vessel occlusion at the level of aneurysmal neck may be another alternative. We present the case of a 10-year-old boy with hemiparesis and multiple cranial nerves paralysis due to severe brainstem compression by a large and growing fusiform basilar aneurysm, who was treated successfully with partial intra-aneurysmal embolization combined with mid-BA occlusion by endovascular techniques.

## Case Report

**Fig. 1:**
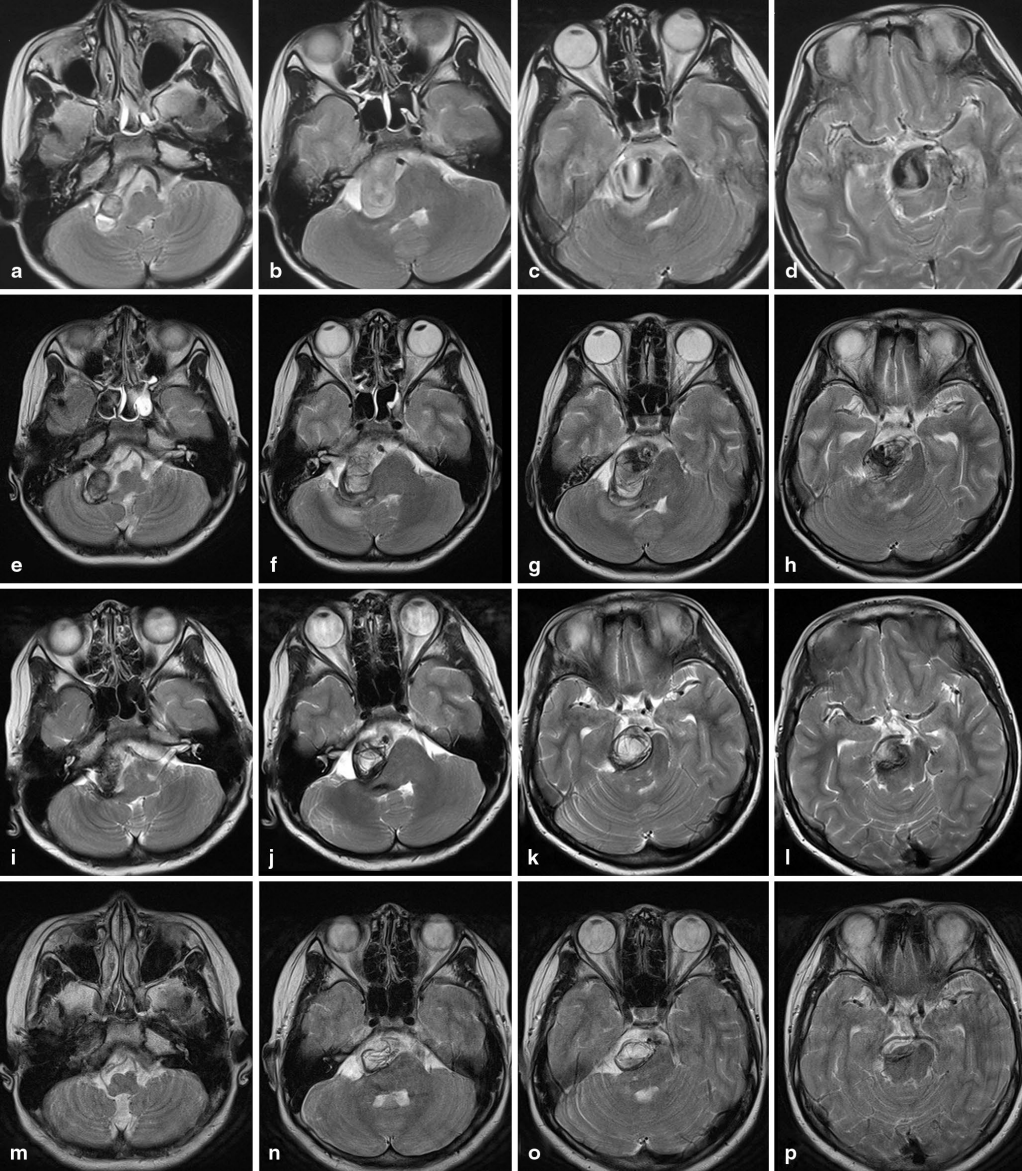
**a**–**d** The magnetic resonance imaging (MRI) T2 revealed a partially thrombosed giant aneurysm of the basilar artery trunk before treatment. **e**–**h** Follow-up MR confirmed the thrombosis of the aneurysm with an increase in the mass effect on the brainstem in the next week after treatment. **i**–**l** The 3-month MRI revealed significant reduction in the size of the aneurysm and in the mass effect on the brain stem. **m**–**p** The 23-month MRI showed further shrinkage of the aneurysm

A 10-year-old boy was admitted to our hospital with progressive vertigo, nausea, and vomiting for 17 days. Neurological examination indicated left hemiparesis and multiple cranial nerves palsy of right third, sixth, seventh, and bilateral ninth, tenth. Computed tomography and magnetic resonance imaging (MRI) revealed a partially thrombosed giant aneurysm of the BA trunk without any sign of bleeding. Mass effect on the brainstem was prominent, and the aneurysm measured 40 mm on MRI (Fig. 1a–d). Selective vertebral with 3-dimensional rotational angiography confirmed the giant fusiform, probably dissecting basilar aneurysm, which originated approximately 0.5 cm distal to the both anterior inferior cerebellar arteries and apparently ended in both posterior cerebral arteries (PCAs), and neither of the superior cerebellar arteries can be found. At bilateral internal carotid artery injection, there was no filling of both PCAs over the circle of Willis. The first digital subtraction angiography was conducted in other hospital; the balloon occlusion test and carotid compression test were not performed. After multidisciplinary discussion, it was decided that the best solution was to overlay two or three Enterprise stent (because it was impossible to obtain a high-density mesh stent, such as Silk or Pipeline) from the proximal BA to PCA P1 segment to divert the blood flow direction and then fill the patent portion of aneurysm with minimum coils, and hoping that the mass effect could be limited to least extent. The alternative treatment based on the collateral circulation checking, if the patient has sufficient collaterals over the posterior communicating arteries, was that the proximal basilar trunk be occluded by coils, if not, a superficial temporal artery to PCA bypass surgery is needed preceding BA occlusion.

**Fig. 2:**
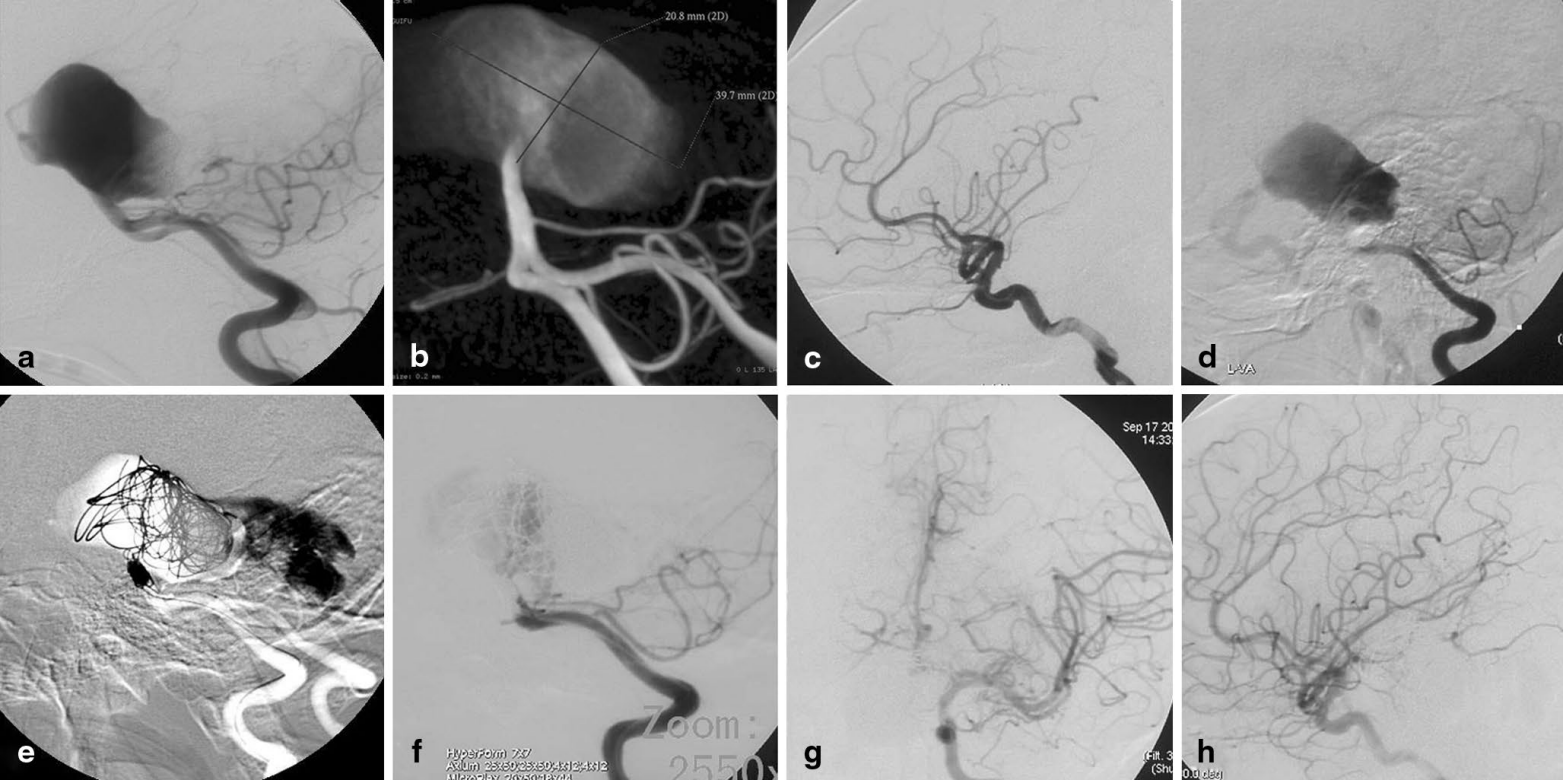
**a** Left vertebral angiography found the giant fusiform, originating approximately 0.5 cm distal to the both anterior inferior cerebellar arteries. **b** 3D measurement of the aneurysm (39.7*20.8 mm). **c** Left posterior communicating artery was not found at the left internal carotid artery injection before treatment. **d** A retrograde filling of the left internal carotid artery was seen on the vertebral artery injection and left carotid compression. **e** The patent portion of aneurysm was partially embolized and then the mid-basilar artery was occluded by coils above the level of the origin of the anterior inferior cerebellar arteries. **f** Final angiographic evaluation confirmed total exclusion of the blood flow from mid-basilar artery to aneurysm. **g–h** Bilateral posterior cerebral arteries were filling through left posterior communicating artery at the left internal carotid artery injection after treatment

**Fig. 3:**
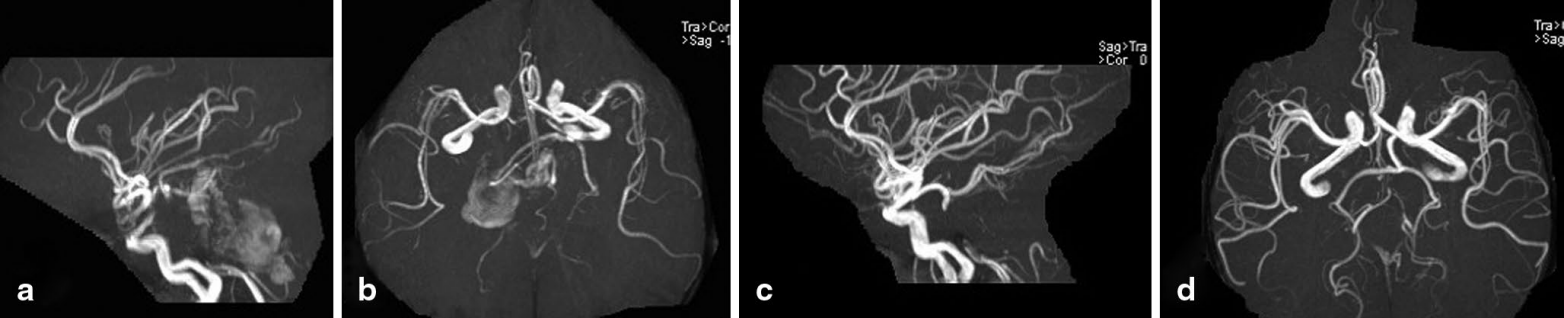
**a**–**b** Magnetic resonance angiography (MRA) at the next week after treatment show the collateral circulation over the left posterior communicating artery supplying bilateral posterior cerebral artery and top of basilar artery. **c**–**d** Follow-up at 23-month MRA shows collaterals and exclude any kind of endoleak situation

The procedure was performed under general anesthesia. The patent portion of aneurysm is 39.7*20.8 mm (Fig. 2a–b). First, an attempt was made to catheterize the distal BA and left PCAs with a microcatheter (Headway 17, MicroVention, Inc. 75 Columbia, Ste A. Aliso Viejo, California 92656, USA) through the aneurysm, but this proved impossible because of the volume of the aneurysm and the pulsations in the sac. Therefore, bilateral carotid compression test was performed separately. A retrograde filling of the left posterior communicating artery (PCoA) and internal carotid artery was seen on the vertebral artery (VA) injection and left carotid compression, proving the functionality of the circulus of Willis on the left (Fig. 2c–d). Then, we decided to partially embolize the patent portion of aneurysm and occlude the BA above both anterior inferior cerebellar arteries. Assistance with temporal occlusion of the proximal BA by Hyperform 7*7 mm balloon (ev3 Inc. 9600 54th Avenue N.Plymouth, MN 55442- 2111 USA), the patent portion of aneurysm was partially embolized with coils (Axium, two 25mm*50 cm, one 20mm*50 cm, and one 18mm*44 cm, ev3 Inc), and then the mid-BA was occluded by coils (Axium, one 4mm*12 cm, hydrocoil, one 3mm*10 cm, and one 2mm*6 cm, ev3 Inc) above the level of the origin of the anterior inferior cerebellar arteries (Fig. 2e). Final angiographic evaluation confirmed total exclusion of the blood flow from BA to aneurysm (Fig. 2f), and bilateral PCAs were filled through left PCoA at the left internal carotid artery injection (Fig. 2g–h). The patient has had aspirin 100 mg and clopidogrel 50 mg for 4 days before treatment. After endovascular therapy, clopidogrel 50 mg were given continuously for 3 days combined with low-molecular-weight heparin calcium injection (GlaxoSmithKline, 0.4 ml, q12h) for 1 week. Follow-up MR in the next week confirmed the thrombosis of the aneurysm with an increase in the mass effect on the brainstem (Fig. 1e–h). Slight aggravation of symptoms was found in the patient after endovascular treatment for 2 weeks. After that, gradual improvement of the neurological deficits was observed, and all symptoms resolved within 3 months. The 3-month MRI revealed significant reduction in the size of the aneurysm and in the mass effect on the brainstem (Fig. 1i–l). MRI at 7- and 23-month showed further shrinkage of the aneurysm (Fig. 1m–p). Magnetic resonance angiography at the next week (Fig. 3a–b) and at 23-month (Fig. 3c–d) show the collateral circulation over the left PCoA supplying bilateral PCA and top of BA, and exclude any kind of endoleak situation.

## Discussion

Giant, fusiform, and serpentine aneurysms, particularly those in the posterior circulation, remain a daunting prospect from both the neurosurgical and endovascular standpoints. Patients with those aneurysms continue to have poor long-term prognosis. Endovascular treatment offers a relatively noninvasive means of approaching posterior fossa aneurysms. However, few reports on endovascular occlusion of the giant BA aneurysms with BA occlusion are published. Hodes et al. [19] and Aymard et al. [20] have described successful treatment of BA aneurysms with balloon occlusion of the BA or vertebrobasilar junction, respectively. In the series by Uda et al. [16], five BA aneurysms were successfully treated by unilateral or bilateral VA coil occlusion. Recently, Jones et al. [21] and Wenderroth et al. [22] have published their cases describing successful endosaccular treatment of giant BA aneurysm with BA trunk occlusion. Endovascular BA occlusion alone without aneurismal packing for blood flow reversal is one alternative treatment for geometrically difficult vertebrobasilar aneurysms either through unilateral or bilateral VA occlusion or selective BA occlusion [23–25].

**Fig. 4:**
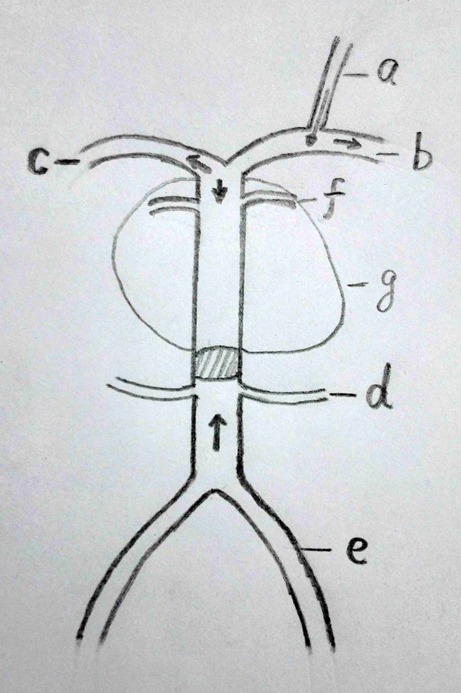
Diagrammatic representation of the reversed basilar artery blood flow following mid-basilar artery occlusion. Inflow occurs via the left posterior communicating artery for bilateral posterior cerebral artery and top of basilar artery. Vertebral arteries supply to bilateral posterior inferior cerebellar arteries. **a** Left posterior communicating artery. **b** Left posterior cerebral artery. **c** Right posterior cerebral artery. **d** Left anterior inferior cerebellar artery. **e** Left vertebral artery. **f** Superior cerebellar artery (maybe absent). **g** Aneurysm

The size of the PCoA to provide sufficient collateral flow to the territory of the occluded vessels is important when the proximal BA was occluded; but in this case, the PCoA is only on the left and small. At first, we attempted to preserve the BA flow by using multiple stent overlay techniques, but this attempt failed, so BA occlusion is the only choice left to prevent the expansion of the aneurysm and alleviate the mass effect on brainstem (Fig. 4). However, little is known about the long-term treatment effect with respect to mass effect on the brainstem or prevention of primary or recurrent subarachnoid hemorrhage. Should the remaining aneurysm lumen after occlusion of the proximal basilar trunk be filled with coils to induce thrombosis? How dense should be the packing of the aneurysm itself? We can not answer these questions accurately. According to Halbach et al. [26], resolution of neurological deficits related to aneurysm mass effect after endosaccular coil treatment is due to resorption of thrombus, clot retraction, and diminished transmitted arterial pulsation. In our case, we occluded the mid-BA for blood flow reversal to diminish the transmitted arterial pulsation from posterior circulation. However, there is no guarantee of complete aneurysmal isolation from the circulation and then thrombosis in the sac. But full embolization of the aneurysm with coils will aggravate the mass effect on the brainstem and reduce the percentage of further shrinkage of the aneurysm. Therefore, we decided to partially embolize the patent portion of the aneurysm. From our limited experience, we think 20 % of packing dense is enough to promote thrombosis in the aneurysm. First, the coils can induce thrombosis in aneurysm sac. Second, rarefaction state of the coils in the sac will be compressed on the resorption stage and then the mass effect will be alleviated. A little aggravation of symptoms was found in the patient after endovascular treatment for 2 weeks. We assume this aggravation of symptoms following endovascular treatment to the mass effect and subsequent perianeurysmal edema was created by thrombus within the aneurysmal sac, but it can be alleviated partially by diminished transmitted arterial pulsation. Considering the perforator branches occlusion and collateral flow network formation maybe occurred in 1 week, we decided to give the patient anticoagulation for 1 week, and we think further platelet antithrombogenic therapy is not necessary for this case. Follow-up MR at the next week confirmed the thrombosis of the aneurysm with an increase in the mass effect on the brainstem, and no ischemic injury can be found in the territory of perforator branches proving the functionality of the left PCOM artery. Recovery of these temporary deficits occurring within 2 weeks after endovascular treatment confirmed our hypothesis. The gradual clinical improvement after 2 weeks presumably reflects cessation of transmitted arterial pulsation and regression of perianeurysmal edema. With the resorption of thrombus and clot retraction, the mass effect on the brainstem was gradually relieved in the following 3 months.

In the future, how to select suitable endovascular treatment for giant fusiform basilar aneurysm is still a question. With the recent introduction of easy-to-place stents and flow diverters, reconstructive endovascular treatment is now possible in most patients with giant fusiform vertebrobasilar aneurysms [27]. But in a recent report of seven patients (six symptomatic, one incidental) with fusiform vertebrobasilar artery aneurysms treated with flow diverters, four patients died, one was severely disabled, and only two did well [28]. Due to the potential risk of perforator ischemia and the risk of very late thrombosis of those flow diverter constructs as well of the risk of remote hemorrhage and late rupture of the aneurysms itself [29, 30], flow diversion treatment by endovascular means is one option but is not necessarily the safest or most definitive treatment modality. For this case, by the current view, the decision to sacrifice vessel distal of the origin of the AICA maybe the most correct treatment.

### Conflict of Interest

The authors declare that there are no actual or potential conflicts of interest in relation to this article.
